# Sensing Viscosity Decoupling and Dynamic Heterogeneity in Tetrabutylammonium Bromide:Polyol Deep Eutectic Solvents

**DOI:** 10.1021/acs.jpcb.6c03764

**Published:** 2026-09-23

**Authors:** Andrew J. Wendel, Gary A. Baker, G. J. Blanchard

**Affiliations:** † 3078 Michigan State University, Department of Chemistry, East Lansing, Michigan 48824-1322, United States; ‡ University of Missouri-Columbia, Department of Chemistry, Columbia, Missouri 65211, United States

## Abstract

We report the reorientation dynamics of the chromophores resorufin and perylene in tetrabutylammonium bromide (TBAB)-based deep eutectic solvent (DES) systems formed with ethylene glycol (EG), 1,3-propanediol (PD), and 1,4-butanediol (BD) across a series of constituent ratios for each DES system. The reorientation dynamics of both chromophores are largely independent of the bulk viscosity of the DESs, consistent with viscosity decoupling and dynamic heterogeneity. This behavior suggests that the characteristic domains sampled by the chromophores are larger than their hydrodynamic volumes. For the polar chromophore resorufin, the results provide evidence for relatively strong interactions with TBA^+^ in the TBAB:PD and TBAB:BD systems, but not in TBAB:EG, suggesting competitive interactions between hydrogen-bond-acceptor-rich domains and hydrogen-bond-donor-rich domains. TBAB-based DESs, therefore, differ from choline chloride-based DESs primarily because TBAB lacks the primary alcohol functionality present in choline chloride.

## Graphical abstract

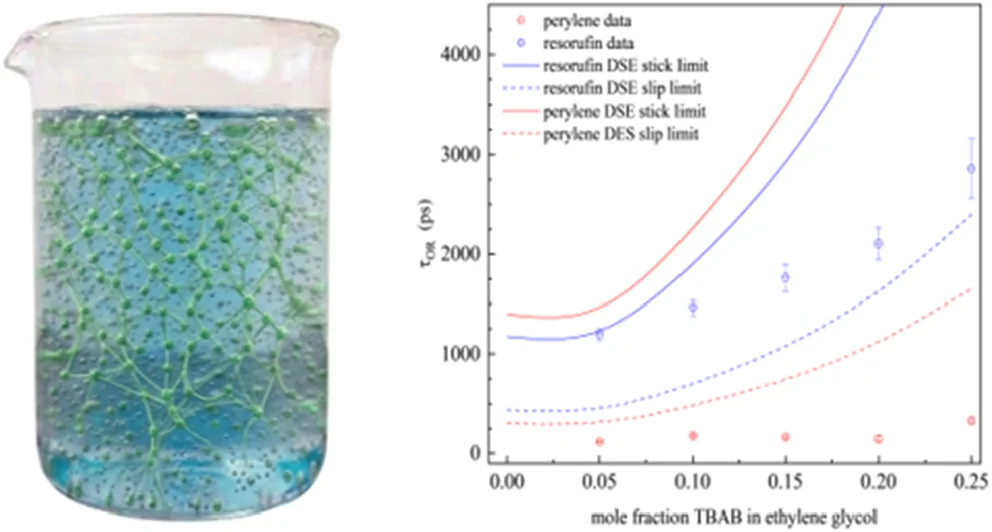

## Introduction

Deep eutectic solvents (DESs) are a class of materials that have attracted much recent attention, for fundamental as well as practical reasons.^1–7^ DESs were discovered relatively recently,^8,9^ and the field has since grown to encompass five classes, with the common property that a mixture of two or more compounds results in a liquid with a melting point significantly lower than the melting points of the pure constituents. The resulting systems have proven to be useful as potential substitutes for molecular solvents because the DES constituents are typically environmentally benign and are readily available. In addition, the constituent ratios can be varied to control physical and chemical properties.^6^ Despite these favorable attributes, there remains much to be explored regarding DES fundamental properties, including the molecular interactions responsible for the anomalously large freezing point depression, and the nanoscale organization that is characteristic of these materials. Part of the challenge to understanding these materials is their typically high viscosity due to strong intermolecular interactions. It is these interactions that also play a deterministic role in their local heterogeneity.

We consider at the outset that the term “DES” refers not to a specific stoichiometric ratio, but rather to a range of stoichiometric ratios of a given system that exhibit a freezing point depression that renders them liquids at room temperature. We use this terminology for consistency with literature extant. For these systems we use the terms mixture(s) and DES(s) interchangeably when referring to the binary systems. We are well aware that the classification of these materials as true DESs remains under discussion as the field grows and evolves. From a practical standpoint, the systems reported here exhibit all the useful characteristics of a DES. We have elaborated on this point in a previous publication.^2^

The archetypal DES is a 1:2 mixture of choline chloride (ChCl) and urea, commonly referred to as reline.^8^ Choline chloride has been widely used as a DES constituent because it contains both quaternary ammonium and primary hydroxyl functionalities, enabling it to participate in binary systems as both a hydrogen bond acceptor (HBA) and donor (HBD), respectively. Although its primary role is typically considered to be that of a HBA, the hydroxyl group can also contribute to the hydrogen-bonding network and influence the local solvation environment. Polyols, including ethylene glycol, glycerol, and the propanediols, have been widely used as HBD constituents because of their high density of hydroxyl functionality and the broad range of physical properties accessible within this class of molecular liquids. Studies of these systems have pointed to the existence of what is termed dynamic heterogeneity, a property where neither of the constituent species behaves in the same manner as the macroscopic bulk material, but rather there exists a dynamic distribution of local high and low viscosity regions.^10–14^ The characterization of such systems is complicated by the rapid exchange of system constituents (both HBD and HBA) between domains and the lack of detailed information on the average domain size(s). To this point, ChCl:polyol DESs have been shown to exhibit a decoupling between bulk viscosity and molecular scale viscosity based on rotational diffusion measurements of neutral and charged chromophores.^2–4^ In the current work, we seek to understand how the structure of tetrabutylammonium bromide (TBAB), as the hydrogen bond acceptor influences local organization and whether this local organization is reflected in the bulk physical properties of the DES.^15–18^ TBAB was chosen because it can serve only as a HBA, in contrast to ChCl, which is both an HBA and HBD. Toward this end, we report here on a series of DESs composed of tetrabutylammonium bromide (TBAB) as the hydrogen bond acceptor and the linear diols ethylene glycol (EG), 1,3-propanediol (PD), and 1,4-butanediol (BD) as their partner hydrogen bond donors, with the HBA:HBD ratio varied systematically in each system. We find that the local environments sampled by both neutral and anionic chromophores are decoupled from the bulk properties of these DESs and are, in all cases, characterized by low apparent viscosity. We also find evidence consistent with an important role for ionic charge in TBAB-based DESs, a feature that is less apparent in choline chloride-based DESs containing the same or structurally similar HBDs. Taken together, these results support the generality of dynamic heterogeneity in DESs and highlight the importance of the choline hydroxyl functionality in governing DES structure and properties.

## Materials and Methods

### DES Preparation

Tetrabutylammonium bromide (TBAB, ≥99.0%, ReagentPlus), ethylene glycol (EG, ≥99%, ReagentPlus), 1,3-propanediol (PD, 98%), and 1,4-butanediol (BD, ≥99%, ReagentPlus) were purchased from MilliporeSigma (St. Louis, MO, USA) and used as received. Three series of TBAB-based DESs were prepared by combining TBAB with one of three linear diol HBDs: EG, PD, or BD. For each series, samples were prepared at TBAB mole fractions, *X*_TBAB_, of 0.05, 0.10, 0.15, 0.20, 0.25, and 0.33. These compositions correspond nominally to diol:TBAB molar ratios of 19:1, 9:1, 5.67:1, 4:1, 3:1, and 2:1, respectively. Selected compositions were excluded from further study because they formed metastable liquids that ultimately solidified at ambient temperature, as indicated by their absence from Table 1. DES samples were prepared using methods reported previously.^3,4^ Briefly, the appropriate masses of the DES components were carefully weighed to an accuracy of ±0.0001 g using an analytical balance (Mettler Toledo LA204E) in a dry 250 mL round-bottom flask. The mixtures were then subjected to rotary evaporation at 65–70 °C for 1 h at 100 rpm. The resulting clear, colorless, homogeneous fluids were transferred to precleaned, oven-dried 40 mL EPA vials containing PTFE-coated stir bars and capped with PTFE-faced silicone rubber septa. The samples were then further dried for at least 24 h at 70 °C on a Schlenk line. Coulometric Karl Fischer (KF) titration indicates water contents of ca. 200 ppm across all DES systems, a level that represents a practical lower limit for polyol-containing DESs dried using conventional methods and can therefore be considered indicative of a scrupulously dry sample. The structures of the DES constituent species are shown in Figure 1.

**Figure 1:**
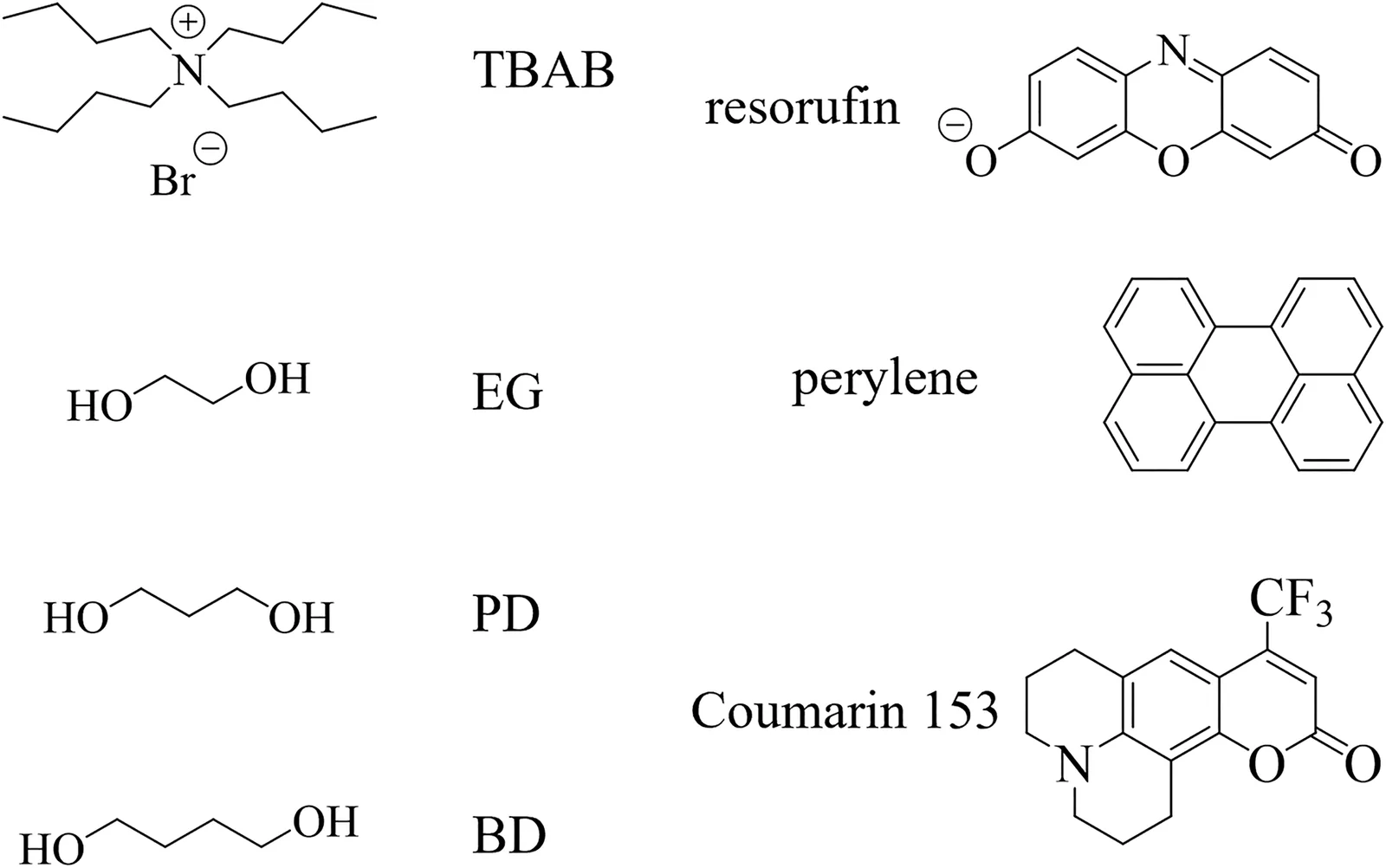
Structures of the DES compounds and fluorescent probes used in this work.

**Table 1:** Densities and Viscosities of DESs^a^,^b^,^c^,^d^

	TBAB:EG		TBAB:PD		TBAB:BD	
*X*_TBAB_	ρ (g/cm^3^)	η (cP)	ρ (g/cm^3^)	η (cP)	ρ (g/cm^3^)	η (cP)
0	1.11^d^	19.6 ± 0.3	1.05^d^	51.2^d^	1.02^d^	91.3^d^
0.05	0.98 ± 0.01	20.5 ± 0.2	0.94 ± 0.02	50.8 ± 1.2	0.92 ± 0.02	81.9 ± 1.5
0.10	1.00 ± 0.04	31.7 ± 1.2	0.92 ± 0.02	68.3 ± 1.4	0.92 ± 0.01	104.5 ± 1.3
0.15	1.05 ± 0.07	48.8 ± 3.2	0.95 ± 0.02	95.4 ± 2.2	0.91 ± 0.02	133.3 ± 3.6
0.20	1.08 ± 0.01	73.5 ± 1.1	0.93 ± 0.03	129.7 ± 4.8		
0.25	1.08 ± 0.03	108.2 ± 3.5	0.94 ± 0.03	186.7 ± 5.6		

a Values for DESs are averages of at least six individual determinations and uncertainties are ±1σ. *T* = 20.0 ± 0.5 °C.

b Hayduk, W.; Malik, V. K., *J. Chem. Eng. Data*, **1971**, *16*, 143–146.

c Zhang, R., Yue, X, Li, B., Yang, J. Wu, Z, Zhang, J., *J. Mol. Liq.*, **2020**, *299*, 112213.

d Kong, L., Li, B., Zhao, L., Zhang, R., Wang, C., *J. Mol. Liq.*, **2021**, *326*, 115107.

### Chromophores

Perylene and resorufin were purchased from Sigma-Aldrich and Coumarin 153 was purchased from Lambda Physik (Figure 1). All chromophores were used as received. Ethanolic 10^–4^ M chromophore stock solutions were used for incorporation into DESs. Aliquots of the stock solutions were evaporated to dryness and then taken up in the DES, to a final chromophore concentration of ∼10^–6^ M.

### Time-Resolved Fluorescence Measurements

Time domain fluorescence measurements were performed using a time-correlated single photon counting (TCSPC) system that has been described in detail previously,^19^ and we provide only a brief recap here. The source laser is a diode-pumped passively mode locked Nd:YVO_4_ laser (Spectra Physics Vanguard) that produces 13 ps pulses at a repetition rate of 82 MHz, with output of 2.5 W average power at 355 and 532 nm. The outputs of this laser are used to excite cavity-dumped dye lasers synchronously. For experiments using perylene, the dye laser (Coherent 701-3 laser, 7220 cavity dumper, 7200 cavity dumper driver) is excited with 355 nm pulses and produces 435 nm output pulses (5 ps FWHM) at a repetition rate of 4.1 MHz with less than 1 mW average power at the sample. For experiments using resorufin, the dye laser is excited with 532 nm pulses and produces 580 nm output pulses (5 ps FWHM) at a repetition rate of 4.1 MHz with less than 1 mW average power at the sample. Sample fluorescence is collected using a reflecting microscope objective (Ealing 40×) and passed through a polarizing cube beam splitter (Newport) to two detection channels, each equipped with a subtractive double monochromator (Spectral Products CM-112) and a microchannel plate photomultiplier detector (Hamamatsu R3809U-50). The signals are sent to the detection electronics (Becker & Hickl SPC-132) and a photodiode (Becker & Hickl PHD-400-N) is used as the reference signal. The instrument response function is 35–40 ps FWHM and the software used for data collection was written in-house using National Instruments LabVIEW software. The sensitivities of the polarized detection channels are matched by equalizing the signal intensities of both detection channels for horizontally polarized excitation.

### Absorbance and Fluorescence Measurements

Absorbance measurements were performed using an Agilent Cary 4000 double beam spectrometer with 1 nm spectral resolution. Fluorescence excitation and emission measurements were performed using a Jobin-Yvon Spex Fluorolog instrument with 1 nm spectral resolution. Peak positions for excitation and emission spectra were determined by fitting the experimental data to Gaussian bands in the same manner as described by Das et al.^20^ using OriginPro 2026 software (OriginLab).

### Viscosity Measurements

Viscosity measurements were performed using Canon-Fenske viscometers (Ace Glass, sizes 200 and 300). Density data were acquired gravimetrically. All measurements were performed at 20 ± 0.5 °C. At least six individual determinations were made and the uncertainties are reported as ± 1σ.

## Results and Discussion

An ongoing challenge in DES research is identifying the structural factors that give rise to nanoscale heterogeneity and determining how the associated dynamics govern both molecular-scale behavior and bulk physicochemical properties.^2–4,11–14,18,21^ Although viscosity decoupling has been observed in multiple DES systems, the details of this decoupling and the underlying chemical basis for its occurrence remain unresolved. In this work, we examine DESs in which one constituent, TBAB, can function only as an HBA. This behavior contrasts with DESs formulated with choline chloride (ChCl), which can serve as both an HBA and, through its hydroxyl group, an HBD. Comparing the similarities and differences between the present TBAB systems and previously reported ChCl-based systems provides insight into the molecular interactions that govern local organization in DESs.

We use the rotational diffusion dynamics of neutral and anionic chromophores to interrogate dynamic heterogeneity in TBAB:polyol DESs. Extracting information about the local environments formed in these systems requires consideration of several factors. A logical starting point is therefore to establish a frame of reference against which the experimental data can be compared.

Chromophore rotational diffusion has proven to be an effective probe of molecular scale organization in dynamic systems. The TCSPC instrument described above produces time-domain polarized fluorescence intensity decays. We acquire data for polarizations parallel and perpendicular to the vertical excitation polarization, designated *I*_*||*_(*t*) and *I*_⊥_(*t*), respectively. These data sets are combined according to eq 1 to produce the induced orientational anisotropy function, *R*(*t*),

1 $$R\left(t\right)=\frac{I_{∥}\left(t\right)-I_{⊥}\left(t\right)}{I_{∥}\left(t\right)+2I_{⊥}\left(t\right)}$$

The functional form of the decay of *R*(*t*) contains the chemical and physical information of interest. *R*(*t*) can, in principle, decay with up to five exponential components, but with the appropriate choice of Cartesian coordinate system, *R*(*t*) will exhibit either a one- or two-component exponential decay, with the decay time constant(s) being related to the Cartesian components of the rotational diffusion constant, *D*_rot_.^22,23^ For all of the TCSPC data reported here, *R*(*t*) is observed to decay as a single exponential, which corresponds to a prolate rotor where the unique rotational axis is coincident with the transition dipole moment axis. Under this condition, only one Cartesian component of *D*_rot_ (*D*_z_) is measured and no explicit information on the ratios of the Cartesian components is available from the data. A comparatively simple and well-established framework for relating the decay time constant of *R*(*t*), τ_OR_, to system properties is through the modified Debye–Stokes–Einstein equation,^24^

2 $$\tau_{OR}=6D_{rot}^{-1}=\frac{\eta \mathrm{Vf}}{k_{B}\mathrm{TS}}$$

As noted above, τ_OR_ is the decay time constant of the induced orientational anisotropy function, *D*_rot_ is the rotational diffusion constant, η is the viscosity of the medium, *V* is the hydrodynamic volume of the reorienting chromophore,^25^ *k*_B_ *T* is the thermal energy term, *f* is the frictional interaction term^26^ and *S* is a shape factor to account for the ellipsoidal shape of the rotor.^27^ This model was developed under multiple assumptions, some of which are accounted for by the terms *f* and *S*. However, when experimental data deviate from the predictions of eq 2, the central assumption is associated with the viscosity term, η: namely, that the bulk viscosity of the medium is equivalent to the local viscosity experienced by the reorienting chromophore. The viscosity of a medium reflects the energetic cost associated with molecular species moving past one another. In neat molecular solvents, the bulk viscosity primarily reflects interactions between like species. In contrast, the interactions governing chromophore rotational diffusion occur between the chromophore and the surrounding medium and therefore involve dissimilar molecular species. The energetics of these interactions can differ from those that determine bulk viscosity. Despite this distinction, the correspondence between η_bulk_ and η_local_ is generally quite good for a wide range of dilute solutions in molecular solvents.^19,23,28^ Thus, the measured orientational relaxation time, τ_OR_ is commonly understood to reflect the local viscosity experienced by the chromophore. Accordingly, it is important first to establish the bulk viscosities of the DES systems examined here.

The bulk viscosities and densities of the DES solvent systems examined in this work are provided in Figure 2 and Table 1. We also report the densities of the DESs, as these values were used in the determination of η. The viscosity data show the expected dependence on HBA content, with viscosity increasing systematically as the TBAB mole fraction increases. In contrast, addition of TBAB to the polyol HBD decreases the density, ρ, of the DES to values lower than those of either pure constituent, including TBAB (ρ_TBAB_ = 1.04 g/cm^3^). Using these viscosity data, we can compare the experimentally measured τ_OR_ times for the DES systems with the values predicted by eq 1. The results of this analysis are summarized in Figure 3.

**Figure 2:**
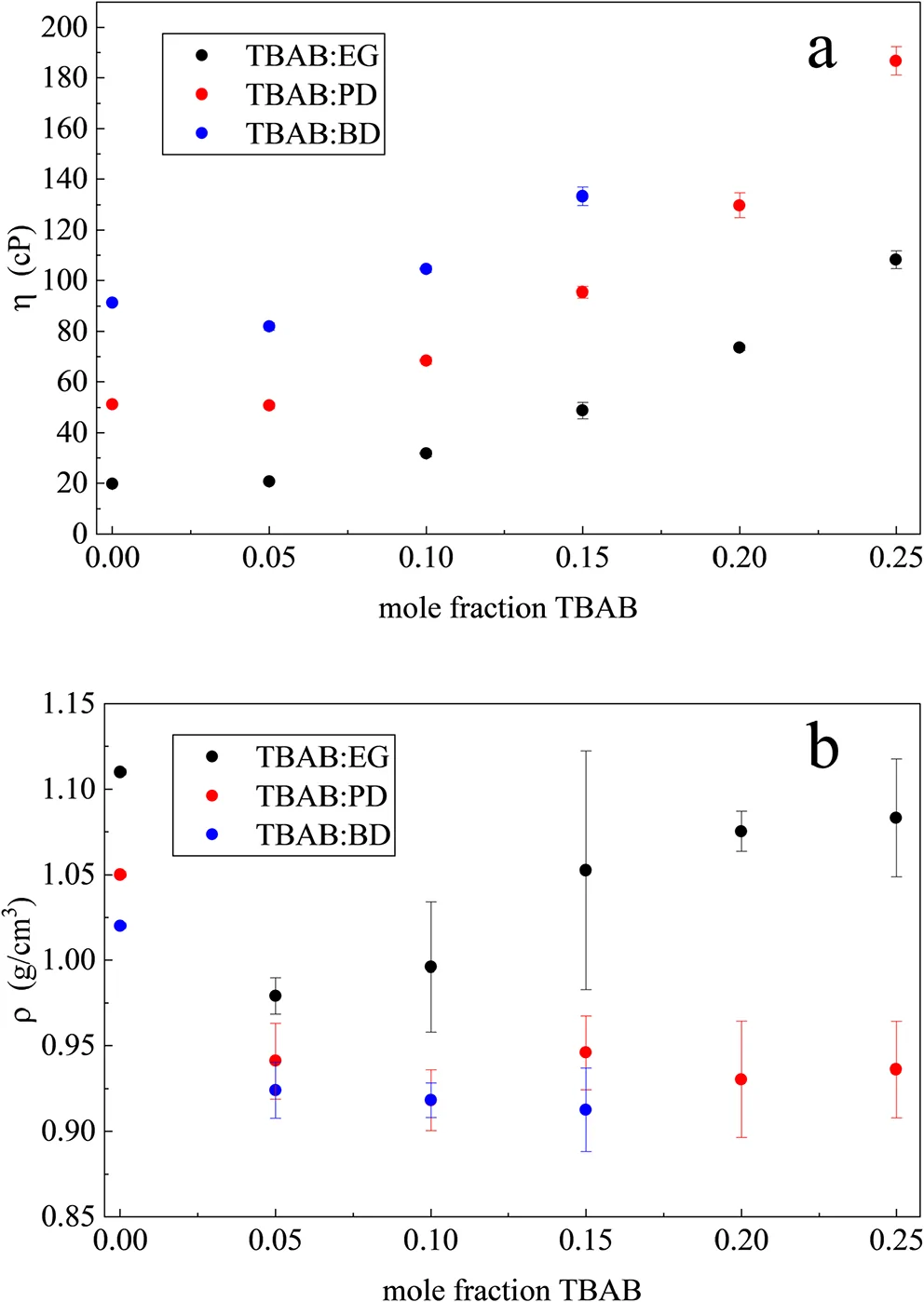
(a) Viscosities (cP) and (b) densities (g/cm^3^) of TBAB-based DES systems as a function of *X*_TBAB_. *T* = 20.0 ± 0.5 °C.

**Figure 3:**
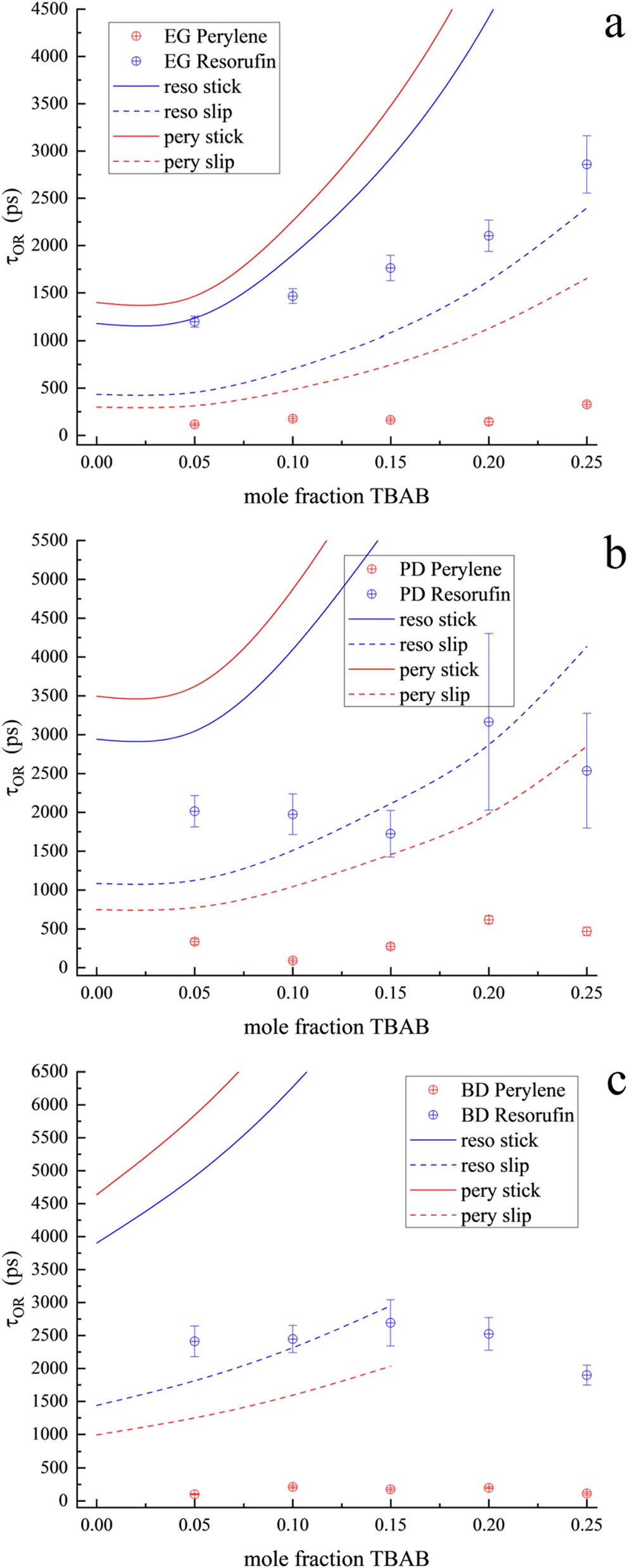
Experimental reorientation times (points) and DSE model predictions (lines) in the stick and slip limits for perylene (red) and resorufin (blue) in (a) TBAB:EG, (b) TBAB:PD, and (c) TBAB:BD as functions of the mole fraction of TBAB, *X*_TBAB_.

The experimental data shown in Figure 3 reveal several interesting features. Most notably, the rotational diffusion time constants depend only weakly on the bulk viscosity of the DES system in all cases. Resorufin exhibits slower orientational relaxation than perylene in each DES, which is qualitatively consistent with the polar character of resorufin and the nonpolar character of perylene. Within the framework of eq 1, this difference is treated through the frictional boundary condition, *f*. For polar chromophores reorienting in polar environments, intermolecular interactions are expected to be relatively strong and are therefore described by the stick-limit, where *f* = 1. In contrast, nonpolar chromophores reorienting in nonpolar media are expected to experience weaker intermolecular interactions and are described by the slip-limit, where 0 < *f* < 1 and the value of *f* depends on the shape of the rotating ellipsoid, as described by *S*.^26,27^ Using the experimental viscosity data in Table 1 and the hydrodynamic volumes of resorufin and perylene, 165 and 225 Å^3^, respectively,^25^ we calculated the predicted τ_OR_ values for each DES system. For the slip limit calculations, the shape factor *S* = 0.68 for resorufin and *S* = 0.78 for perylene, based on molecular dimensions determined using molecular mechanics calculations. Because both chromophores reorient as prolate rotors (*vide infra*), the slip limit frictional boundary terms are *f*_slip_ = 0.369 for resorufin and *f*_slip_ = 0.214 for perylene.^26,29^ These predictions are shown in Figure 3 for both the stick-limit, represented by solid lines, and the slip-limit, represented by dashed lines. As expected, the DSE model predicts increasing τ_OR_ with increasing viscosity for all systems examined, and the slip-limit predictions yield faster relaxation than the corresponding stick-limit predictions.

Comparison of the experimental data with the model predictions shows that, in all cases, perylene reorients much faster than predicted by eq 1 and exhibits dynamics that are essentially independent of bulk viscosity. Resorufin displays reorientation dynamics that fall between the stick and slip limits at the lowest TBAB mole fractions for all HBDs. For the PD- and BD-based DESs, however, the resorufin dynamics become more consistent with sub-slip behavior at higher TBAB mole fractions. Although a weak viscosity dependence is observed for resorufin reorientation in TBAB:EG, no comparable trend is evident in either TBAB:PD or TBAB:BD. Together, these results provide important insight into the local environments and dynamical heterogeneity of these DES systems.

It is useful to consider these results in terms of how each chromophore interacts with its local environment. The pronounced viscosity independence of the reorientation dynamics indicates that the local environment sensed by each chromophore does not change substantially upon addition of TBAB to the polyols. Similar behavior has been observed in other systems, including ChCl-based DESs,^2–4^ and is commonly described as viscosity decoupling.^4,11–14,21^ These data demonstrate that the DES systems are not homogeneous, consistent with observations for the more extensively studied ChCl-based DESs. Their local organization can instead be understood in the context of dynamic heterogeneity, in which the chromophores are sequestered within domains that are sufficiently larger than the chromophores themselves to permit rotational motion without significant sensitivity to the domain interfaces. The results for perylene are particularly instructive. In TBAB:EG, τ_OR_ for perylene is essentially constant from *X*_TBAB_ = 0.05 to 0.20, with a value of approximately 150 ps. This time constant corresponds to a stick-limit local viscosity of ∼10 cP, or approximately the bulk viscosity of neat EG under slip-limit conditions. We do not take this result as evidence for sequestration of perylene into EG-rich domains, however, because perylene behaves similarly in TBAB:PD and TBAB:BD. Rather, the perylene data across all DESs examined here suggest that perylene interacts preferentially with the alkyl-chain regions of TBAB-rich domains, consistent with its nonpolar character.^28^ For resorufin, the interactions are expected to differ because of its greater polarity and its ionic character in the dissociated form (*vide infra*). Although τ_OR_ for resorufin is characteristically slower than that of perylene in all DESs, the experimental τ_OR_ values are near or below the slip-limit predictions. For TBAB:PD and TBAB:BD, the resorufin reorientation dynamics are functionally independent of bulk viscosity. This behavior suggests relatively strong interactions with the TBA^+^ cation, consistent with ionic association. However, resorufin is also soluble in polyols, allowing it to interact with both polar HBD-rich regions and less polar HBA-rich regions. In all cases, the local domains occupied by the chromophores appear to be largely independent of DES composition, consistent with dynamic heterogeneity in these systems.

While the reorientation data are consistent with dynamic heterogeneity, they do not prove its existence. One way to evaluate the existence of persistent local heterogeneity is to examine whether there is any excitation energy-dependence to chromophore emission, or any emission energy-dependence to the chromophore excitation spectrum. These two phenomena have been reported previously through red edge excitation shift (REES) and blue edge emission shift (BEEmS) measurements.^20,30–32^ These steady-state fluorescence excitation and emission measurements provide insight into environmental heterogeneity on time-scales that persists for times the order of chromophore fluorescence lifetime. The absorption and emission spectra of fluorescent organic molecules in the liquid phase are inhomogeneously broadened, and there are multiple chromophore local environments that differ very little in energy of interaction and typically exchange rapidly. As a result, ultrafast time-resolved spectroscopic techniques are required to spectroscopically probe the distribution of local environments and their exchange time, which is thought to be on the order of the Debye relaxation time of the medium. For liquid phase media that are characterized by a distinct distribution of local environments, such as those described by dynamic heterogeneity, however, the persistence time of the domains can be much longer. For such systems, where the persistence time of the domains is similar to or longer than the fluorescence lifetime of the chromophore, it is possible to probe such heterogeneity using steady-state spectroscopic methods. We have used the chromophore Coumarin 153 (C153) to perform REES and BEEmS measurements of TBAB:EG (Figure 4), TBAB:PD (Figure 5) and TBAB:BD (Figure 6) for *X*_TBAB_ = 0.1 and 0.2 for each system. The excitation and emission wavelength dependencies (Figures 4e,f, 5e,f, and 6e,f) reveal behavior consistent with dynamic heterogeneity, and the prominence of these features increases with increasing system viscosity. Notably, the excitation and emission peak position change least for TBAB:EG with *X*_TBAB_ = 0.1, increasing for *X*_TBAB_ = 0.2. We understand this observation in the context of the relative values of the solvent relaxation time (∼100 ps)^33^ relative to the C153 fluorescence lifetime (∼1.5 ns).^34^ The analogous data for TBAB:PD (Figure 5e and f) and TBAB:BD (Figure 6e and f) show more pronounced wavelength-dependent peak maxima, which we attribute to the longer environment relaxation times, although these quantities remain to be quantified.

**Figure 4:**
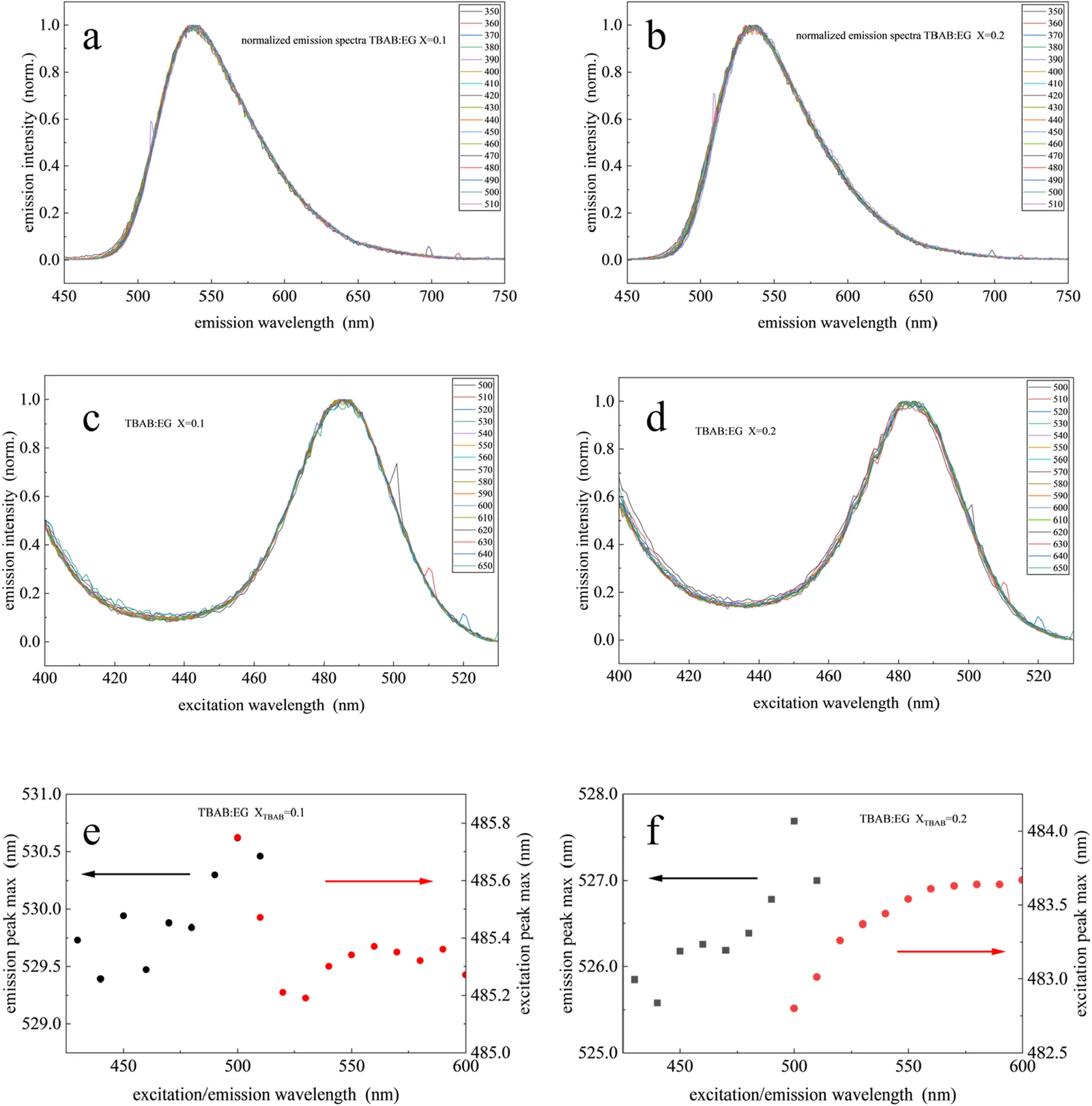
(a and b) Normalized emission spectra of C153 in TBAB:EG as a function of excitation wavelength (indicated in the right panel of each figure) for *X*_TBAB_ = 0.1 (a) and *X*_TBAB_ = 0.2 (b). (c and d) Normalized excitation spectra of C153 as a function of emission collection wavelength (indicated in the right panel of each figure) for *X*_TBAB_ = 0.1 (c) and *X*_TBAB_ = 0.2 (d). (e and f) Emission peak max as a function of excitation wavelength (black) and excitation peak max as a function of emission collection wavelength (red) for *X*_TBAB_ = 0.1 (e) and *X*_TBAB_ = 0.2 (f).

**Figure 5:**
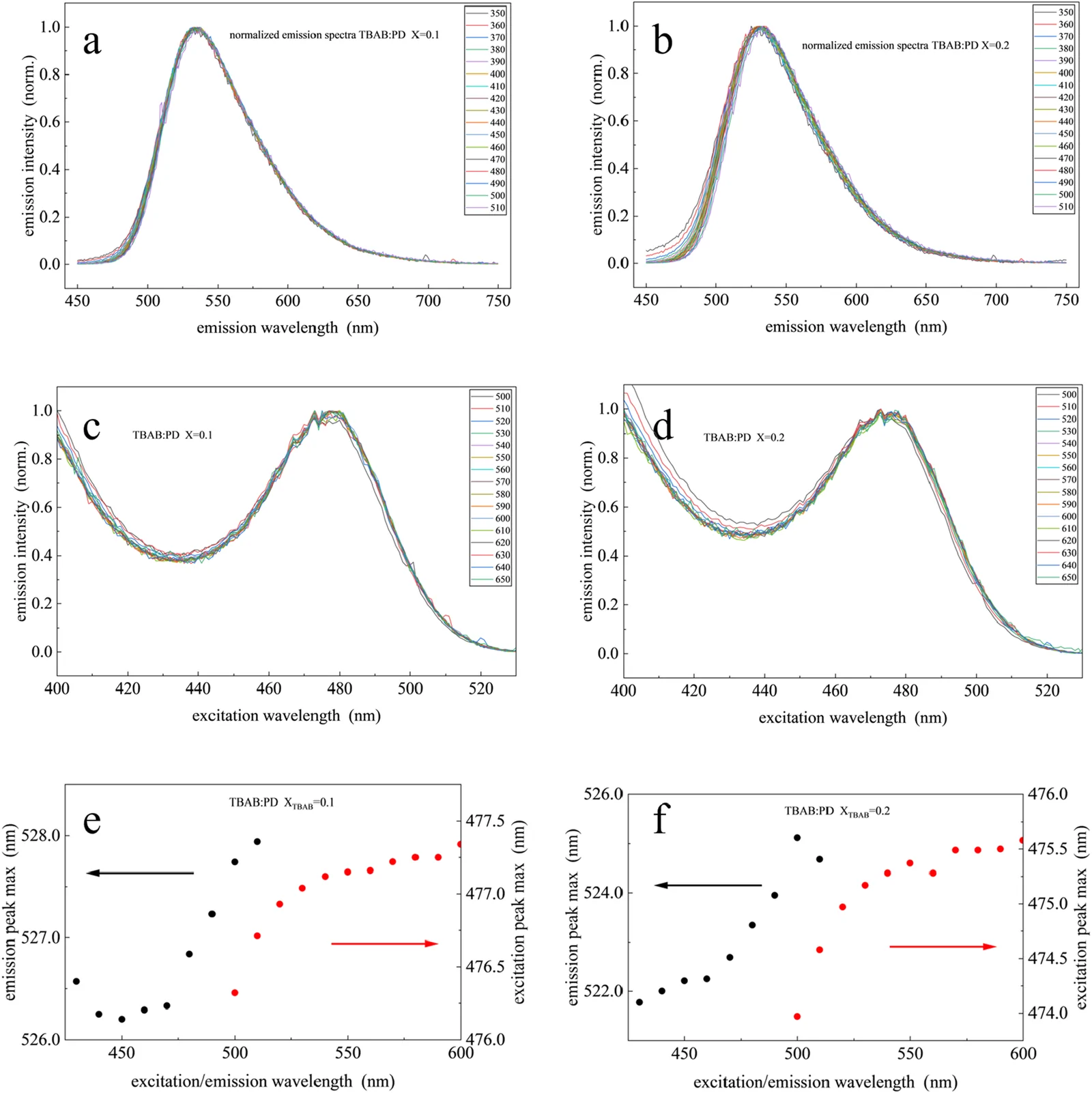
(a and b) Normalized emission spectra of C153 in TBAB:PD as a function of excitation wavelength (indicated in the right panel of each figure) for *X*_TBAB_ = 0.1 (a) and *X*_TBAB_ = 0.2 (b). (c and d) Normalized excitation spectra of C153 as a function of emission collection wavelength (indicated in the right panel of each figure) for *X*_TBAB_ = 0.1 (c) and *X*_TBAB_ = 0.2 (d). (e and f) Emission peak max as a function of excitation wavelength (black) and excitation peak max as a function of emission collection wavelength (red) for *X*_TBAB_ = 0.1 (e) and *X*_TBAB_ = 0.2 (f).

**Figure 6:**
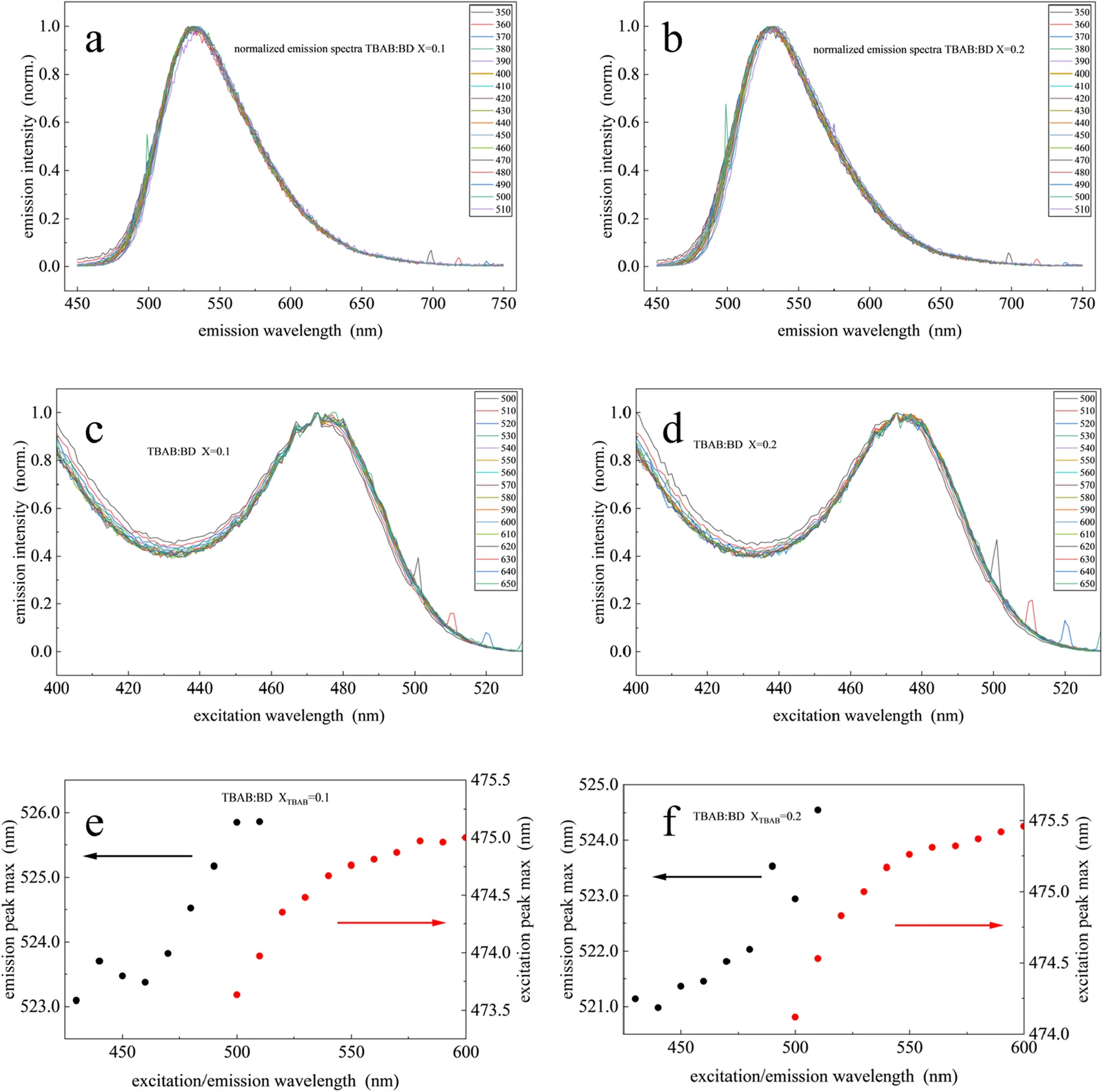
(a and b) Normalized emission spectra of C153 in TBAB:BD as a function of excitation wavelength (indicated in the right panel of each figure) for *X*_TBAB_ = 0.1 (a) and *X*_TBAB_ = 0.2 (b). (c and d) Normalized excitation spectra of C153 as a function of emission collection wavelength (indicated in the right panel of each figure) for *X*_TBAB_ = 0.1 (c) and *X*_TBAB_ = 0.2 (d). (e and f) Emission peak max as a function of excitation wavelength (black) and excitation peak max as a function of emission collection wavelength (red) for *X*_TBAB_ = 0.1 (e) and *X*_TBAB_ = 0.2 (f).

The apparent ionic interactions between TBA^+^ and the resorufin anion are consistent with two additional observations. First, the behavior of the cationic chromophore Oxazine 725 in these DES systems differs substantially from that observed in ChCl-based DESs. Specifically, the fluorescence lifetime of Oxazine 725 is approximately 2-fold shorter in the TBAB-based DESs than in ChCl-based DESs.^2–4^ We attribute this decrease to quenching of the cationic chromophore by the comparatively high concentration of bromide present in these systems. The shortened lifetime of Oxazine 725 (∼1.1 ns for all systems reported here, see Tables S1–S3) precluded its use for τ_OR_ measurements in this work. The second observation consistent with association between resorufin and TBA^+^ comes from the resorufin absorbance data shown in Figure 7. In TBAB:EG (Figure 7 black trace), the dominant feature is the S_1_ ← S_0_ transition of the dissociated resorufin anion, with an absorption maximum at 585 nm and an emission maximum at 601 nm. The spectral properties of resorufin are known to depend sensitively on whether the anion interacts with cationic species.^35^ Interaction of the anionic chromophore with a charged species, when sufficiently strong to perturb chromophore conjugation, gives rise to a blue absorption band with a substantially decreased fluorescence quantum yield. This blue band is evident for resorufin in TBAB:PD (Figure 7 red trace) and TBAB:BD (Figure 7 blue trace). In these spectra, the absorption and emission profiles are normalized to equal intensities for the deprotonated fraction in each DES. The τ_OR_ data reported in Figure 3 are spectroscopically selective for the deprotonated form because of the excitation and emission collection wavelengths used. A clear implication of these data is that, in TBAB:EG, interactions with polar EG-rich regions sequester the resorufin anion away from the TBA^+^ cation sufficiently to preclude observation of the associated form. Consistent with this interpretation, the τ_OR_ data for TBAB:EG exhibit the most pronounced viscosity dependence among the DES systems examined here. In contrast, for TBAB:PD and TBAB:BD, where significant interaction between resorufin and TBA^+^ is observed spectroscopically, the τ_OR_ data are essentially viscosity independent.

**Figure 7:**
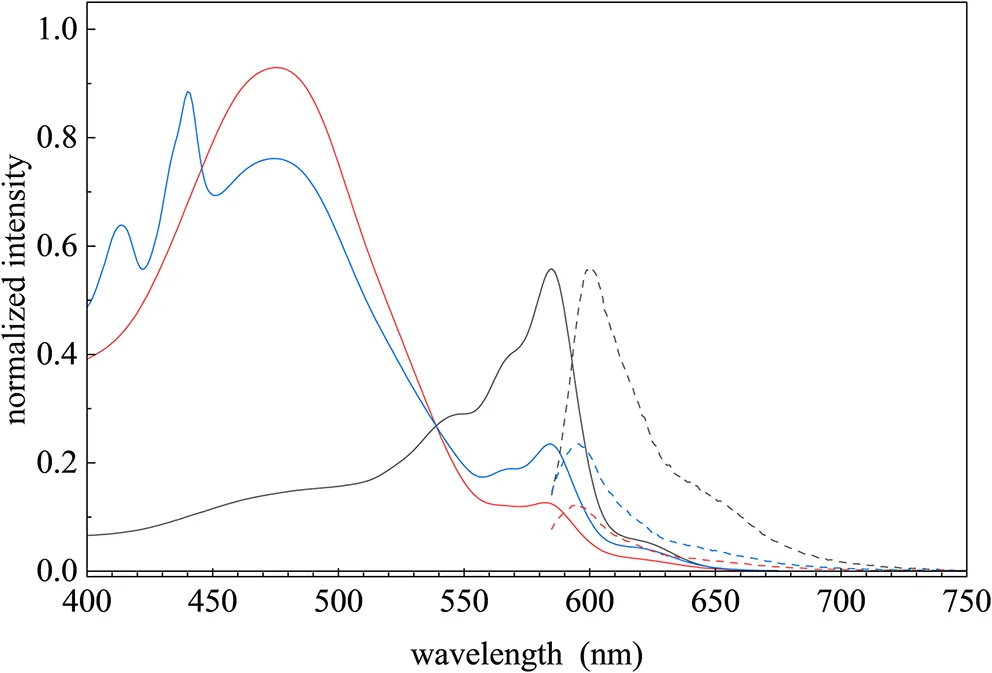
Normalized absorbance (solid lines) and emission (dashed lines) spectra of resorufin in 1:3 TBAB:EG (black), TBAB:PD (red) and TBAB:BD (blue). Emission spectra are normalized to the S_1_ ← S_0_ transition of the resorufin anion.

Finally, it is useful to compare the TBAB-based DES data with analogous results reported for ChCl-based DESs. In ChCl-based systems, the rotational diffusion of charged chromophores exhibits a clear change in behavior near *X*_ChCl_ ∼ 0.17,^2–4^ whereas no analogous discontinuity is evident in the TBAB-based DES data shown in Figure 3. In those earlier studies, this change in reorientation dynamics was suggested to coincide with the true eutectic composition of the DESs. The absence of a comparable feature in the TBAB-based systems suggests that the present reorientation measurements are less sensitive to the local organization responsible for the eutectic point, likely because of the distinct local environments in which the chromophores are sequestered. It is also likely that the terminal hydroxyl functionality of ChCl plays an important role in chromophore localization in ChCl-based DESs, whereas this interaction is absent in the TBAB-based systems.

## Conclusions

We have evaluated the reorientation dynamics of two chromophores, anionic resorufin and neutral perylene, in the TBAB-based DESs TBAB:EG, TBAB:PD, and TBAB:BD over a series of constituent ratios. In all three DES families, the chromophore reorientation dynamics are strongly decoupled from the bulk viscosity, consistent with dynamic heterogeneity and with local domains that are sufficiently large relative to the chromophores to permit rotational motion without substantial sensitivity to domain interfaces. Although these measurements do not allow a quantitative estimate of domain size, they clearly indicate that the local environments sampled by the chromophores are not governed directly by the macroscopic viscosity. The perylene data suggest preferential interaction with nonpolar alkyl-chain regions of TBAB-rich domains, whereas the resorufin data reveal more complex behavior arising from competitive interactions with polar HBD-rich regions and less polar, ion-rich HBA domains. For resorufin, spectroscopic evidence indicates association with TBA^+^ in TBAB:PD and TBAB:BD, but not in TBAB:EG, highlighting the role of HBD identity in mediating chromophore localization. Comparison with analogous ChCl-based DESs suggests that TBAB-based systems differ primarily because TBAB lacks the terminal hydroxyl functionality present in ChCl, which appears to play an important role in organizing local solvation environments. Future studies using asymmetric quaternary ammonium HBAs and systematically varied HBD structures should provide a more complete understanding of how constituent functionality controls nanoscale organization, viscosity decoupling, and solute-specific local environments in DESs.

## Supplementary Material



## Notes

Tabulated values of fluorescence lifetime and anisotropy decay data; example data sets and fitting information for perylene and resorufin (PDF)
