# Supplementary material for: Sensing Viscosity Decoupling and Dynamic Heterogeneity in Tetrabutylammonium Bromide:Polyol Deep Eutectic Solvents
Source: J Phys Chem B. 2026 Sep 23;130(40):10551–60. doi: 10.1021/acs.jpcb.6c03764 (PMC13656730; doi:10.1021/acs.jpcb.6c03764)

Supporting Information

Sensing Viscosity Decoupling and Dynamic Heterogeneity in Tetrabutylammonium Bromide:Polyol Deep Eutectic Solvents

Andrew J. Wendel,[1] Gary A. Baker[2] and G. J. Blanchard[1,*]

[1] Michigan State University, Department of Chemistry, East Lansing, MI 48824-1322 USA
[2] University of Missouri-Columbia, Department of Chemistry, Columbia, MO 65211 USA

Contents Page

Table S1. Fluorescence lifetimes of chromophores used in this work for TBAB:EG……………….S1

Table S2. Fluorescence lifetimes of chromophores used in this work for TBAB:PD……………….S1

Table S3. Fluorescence lifetimes of chromophores used in this work for TBAB:BD……………….S1

Table S4. Anisotropy decay times perylene and resorufin in TBAB:EG……………………………S2

Table S5. Anisotropy decay times perylene and resorufin in TBAB:PD……………………………S2

Table S6. Anisotropy decay times perylene and resorufin in TBAB:BD……………………………S2

Figure S1. Nonlinear Curve Fit (ExpDecay1) for one data set of Resorufin in 1:3 TBAB:PD……..S4

Figure S2. Fitted Curves and Residuals Plot……………………………………………………..S6

Figure S8. Fitted Curves and Residuals Plots……………………………………………………….S9

Figure S9. Nonlinear Curve Fit (ExpDecay1) for one data set of Perylene in Figure S10. Fitted Curves and Residuals Plots…………………………………………………S12Table S10. Fit of perylene anisotropy decay data……………………… …………………………S13

Figure S11. Fitted Curves and Residuals Plots………………………………………………….S14

* Author to whom corresponding should be addressed. Email: blanchard@chemistry.msu.edu, Tel: +1 517 353 1105

Table S1. Fluorescence lifetimes of chromophores used in this work for TBAB:EG. The lifetimes for Oxazine 725 are included, although reorientation dynamics for this chromophore were not used due to the comparatively short lifetime. Lifetimes are in ps and uncertainties are ± 1σ for at least three individual determinations.

X_{TBAB}	Oxazine 725	Perylene	Resorufin
0.33	1249 ± 10	2651 ± 62	2544 ± 36
0.25	1010 ± 6	3194 ± 120	2484 ± 38
0.20	995 ± 7	1940 ± 11	2394 ± 36
0.15	954 ± 7	4384 ± 93	2676 ± 47
0.10	899 ± 5	2426 ± 19	2362 ± 29
0.05	912 ± 6	2234 ± 13	2641 ± 37

Table S2. Fluorescence lifetimes of chromophores used in this work for TBAB:PD. The lifetimes for Oxazine 725 are included, although reorientation dynamics for this chromophore were not used due to the comparatively short lifetime. Lifetimes are in ps and uncertainties are ± 1σ for at least three individual determinations.

X_{TBAB}	Oxazine 725	Perylene	Resorufin
0.33	1299 ± 11	2106 ± 39	2475 ± 46
0.25	1166 ± 9	2382 ± 20	2340 ± 34
0.20	1110 ± 8	2149 ± 44	2355 ± 37
0.15	1127 ± 8	2397 ± 16	2285 ± 38
0.10	1072 ± 8	2536 ± 17	2218 ± 28
0.05	1045 ± 7	2598 ± 22	2154 ± 28

Table S3. Fluorescence lifetimes of chromophores used in this work for TBAB:BD. The lifetimes for Oxazine 725 are included, although reorientation dynamics for this chromophore were not used due to the comparatively short lifetime. Lifetimes are in ps and uncertainties are ± 1σ for at least three individual determinations.

X_{TBAB}	Oxazine 725	Perylene	Resorufin
0.25	841 ± 6	2255 ± 15	2629 ± 41
0.20	877 ± 5	3335 ± 145	2413 ± 36
0.15	1168 ± 8	2116 ± 14	2347 ± 28
0.10	1177 ± 9	2193 ± 16	2106 ± 28
0.05	1124 ± 8	2300 ± 17	2099 ± 27

Table S4. Anisotropy decay times perylene and resorufin in TBAB:EG. Time constants τ_{OR} are in ps and uncertainties are ± 1σ for at least three individual determinations.

X_{TBAB}	Perylene	Resorufin
0.33	350 ± 22	3245 ± 431
0.25	328 ± 24	2858 ± 303
0.20	144 ± 30	2103 ± 166
0.15	164 ± 19	1763 ± 134
0.10	176 ± 21	1465 ± 78
0.05	116 ± 14	1195 ± 58

Table S5. Anisotropy decay times perylene and resorufin in TBAB:PD. Time constants τ_{OR} are in ps and uncertainties are ± 1σ for at least three individual determinations.

X_{TBAB}	Perylene	Resorufin
0.33	607 ± 32	2058 ± 873
0.25	468 ± 55	2536 ± 738
0.20	617 ± 50	3166 ± 1137
0.15	275 ± 33	1725 ± 300
0.10	92 ± 23	1976 ± 261
0.05	336 ± 36	2015 ± 201

Table S6. Anisotropy decay times perylene and resorufin in TBAB:BD. Time constants τ_{OR} are in ps and uncertainties are ± 1σ for at least three individual determinations.

X_{TBAB}	Perylene	Resorufin
0.25	113 ± 25	1899 ± 151
0.20	199 ± 18	2524 ± 248
0.15	176 ± 24	2692 ± 349
0.10	212 ± 21	2447 ± 207
0.05	101 ± 11	2411 ± 234

Figure S1. Nonlinear Curve Fit (ExpDecay1) for one data set of Resorufin in 1:3 TBAB:PD

Raw data

Parameters

		Value	Standard Error	t-Value	Prob>|t|	Dependency
lifetime	y0	365.8099	0	--	--	0
	x0	1230	0	--	--	0
	A1	11481.18384	19.96929	574.94204	0	0.55731
	t1	3517.29387	9.05402	388.47879	0	0.55731

Reduced Chi-sqr = 29340.077905
COD(R^2) = 0.99627920477448
Iterations Performed = 3
Total Iterations in Session = 3
Fit converged. Chi-Sqr tolerance value of 1E-9 was reached.
Some parameter values were fixed.
Standard Error was scaled with square root of reduced Chi-Sqr.

Statistics

	lifetime
Number of Points	846
Degrees of Freedom	844
Reduced Chi-Sqr	29340.0779
Residual Sum of Squares	2.4763E7
R-Square (COD)	0.99628
Adj. R-Square	0.99627
Fit Status	Succeeded(100)

Fit Status Code :
100 : Fit converged. Chi-Sqr tolerance value of 1E-9 was reached.

Summary

	y0		x0		A1		t1		Statistics	
	Value	Standard Error	Value	Standard Error	Value	Standard Error	Value	Standard Error	Reduced Chi-Sqr	Adj. R-Square
lifetime	365.8099	0	1230	0	11481.18384	19.96929	3517.29387	9.05402	29340.0779	0.99627

ANOVA

		DF	Sum of Squares	Mean Square	F Value	Prob>F
lifetime	Regression	2	2.46518E10	1.23259E10	420105.06404	<0.0001
	Residual	844	2.4763E7	29340.0779		
	Uncorrected Total	846	2.46766E10			
	Corrected Total	845	6.6553E9			

lifetime: At the 0.05 level, the fitting function is significantly better than the function y=0.

Figure S2. Fitted Curves and Residuals Plot

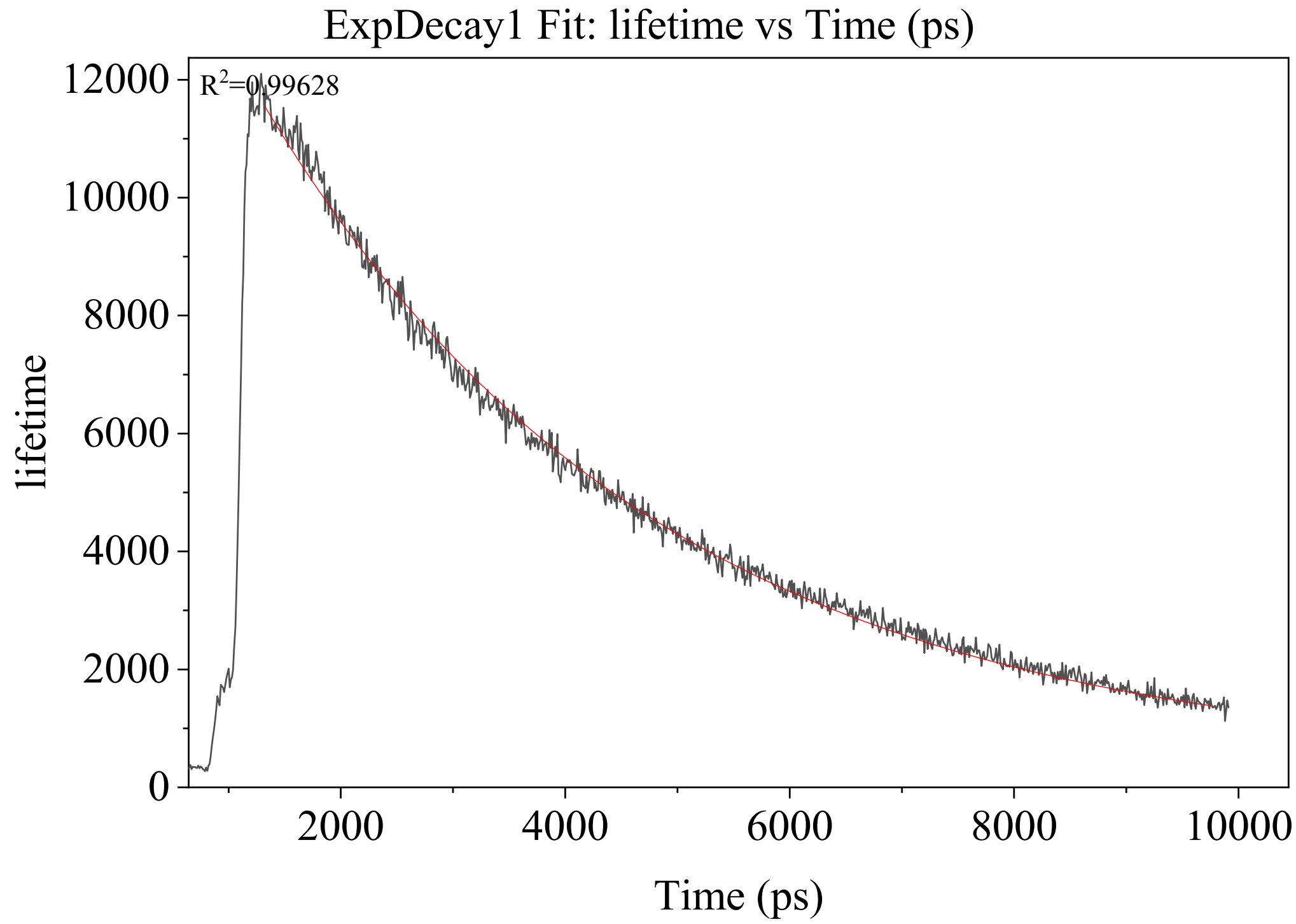

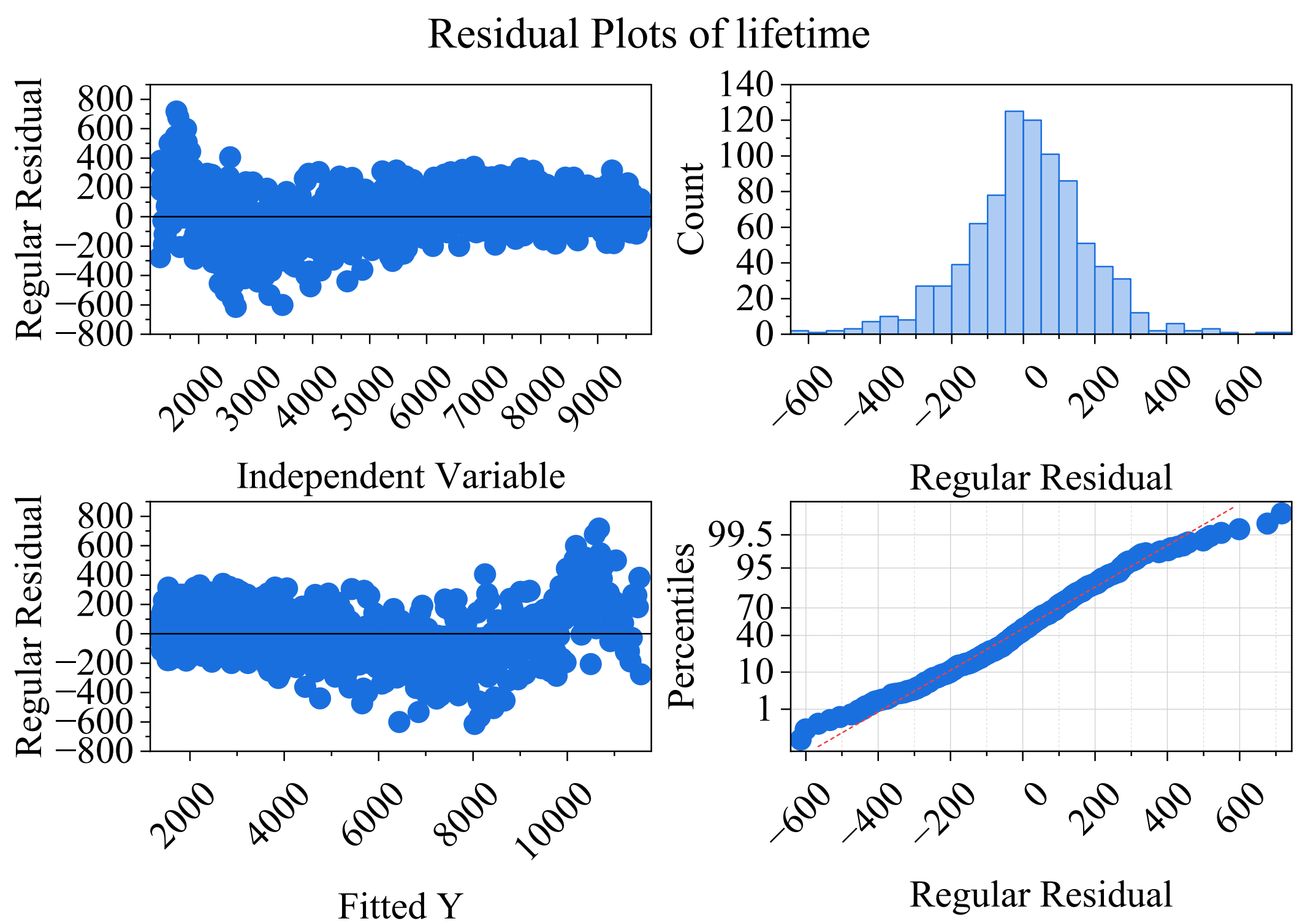

Parameters

		Value	Standard Error	t-Value	Prob>|t|	Dependency
anisotropy	y0	0	0	--	--	0
	x0	1230	0	--	--	0
	A1	0.22552	0.00311	72.5106	0	0.54671
	t1	2677.71061	52.05814	51.43693	8.49191E-257	0.54671

Reduced Chi-sqr = 5.49623479068E-4

COD(R^2) = 0.85681627397992

Iterations Performed = 4

Total Iterations in Session = 4

Fit converged. Chi-Sqr tolerance value of 1E-9 was reached.

Some parameter values were fixed.

Standard Error was scaled with square root of reduced Chi-Sqr.

Statistics

	anisotropy
Number of Points	808
Degrees of Freedom	806
Reduced Chi-Sqr	5.49623E-4
Residual Sum of Squares	0.443
R-Square (COD)	0.85682
Adj. R-Square	0.85664
Fit Status	Succeeded(100)

Fit Status Code :

100 : Fit converged. Chi-Sqr tolerance value of 1E-9 was reached.

Summary

	y0		x0		A1		t1		Statistics	
	Value	Standard Error	Value	Standard Error	Value	Standard Error	Value	Standard Error	Reduced Chi-Sqr	Adj. R-Square
anisotropy	0	0	1230	0	0.22552	0.00311	2677.71061	52.05814	5.49623E-4	0.85664

ANOVA

		DF	Sum of Squares	Mean Square	F Value	Prob>F
anisotropy	Regression	2	6.37512	3.18756	5799.53826	<0.0001
	Residual	806	0.443	5.49623E-4		
	Uncorrected Total	808	6.81812			
	Corrected Total	807	3.0939			

anisotropy: At the 0.05 level, the fitting function is significantly better than the function y=0.

Figure S8. Fitted Curves and Residuals Plots

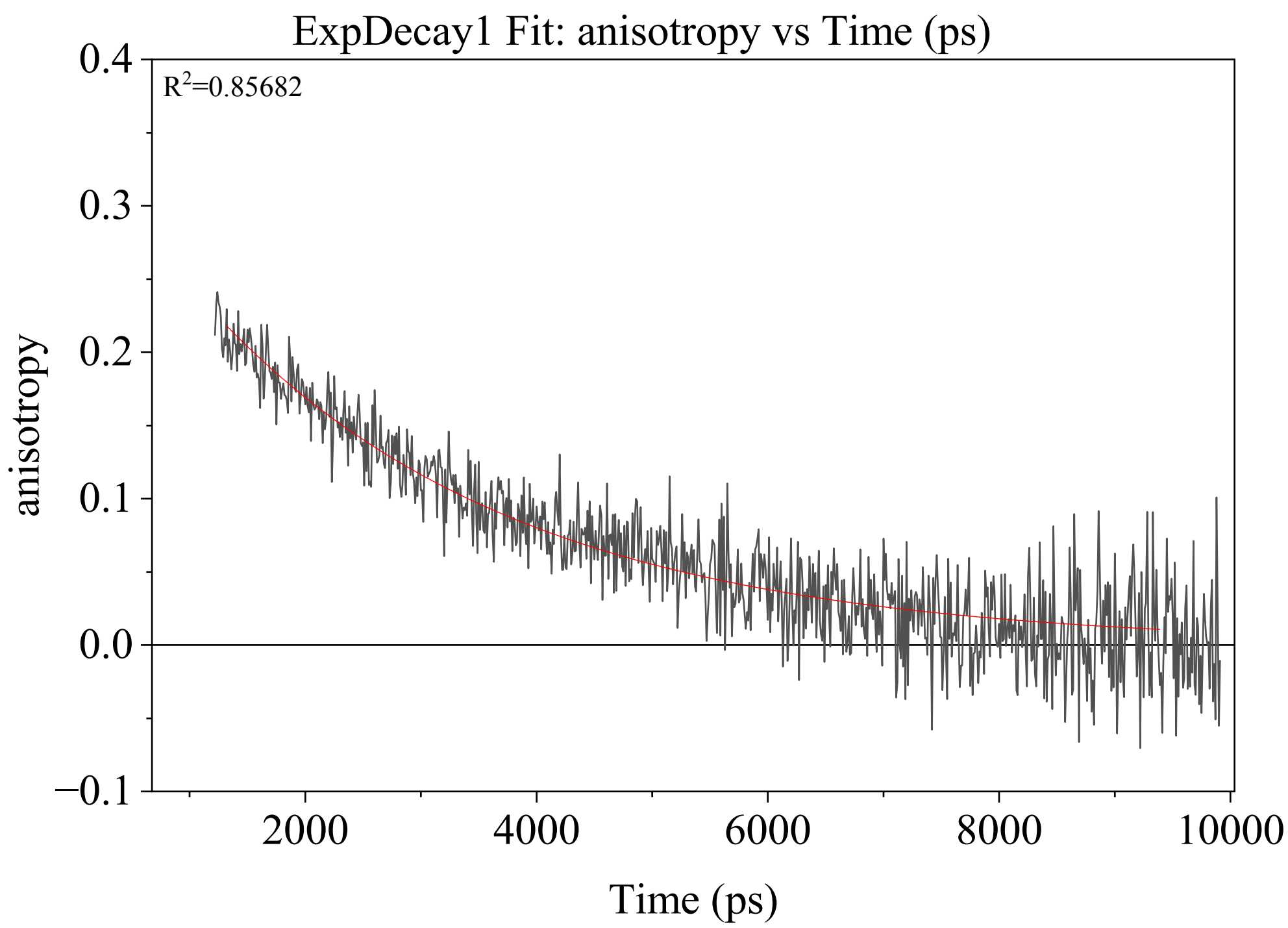

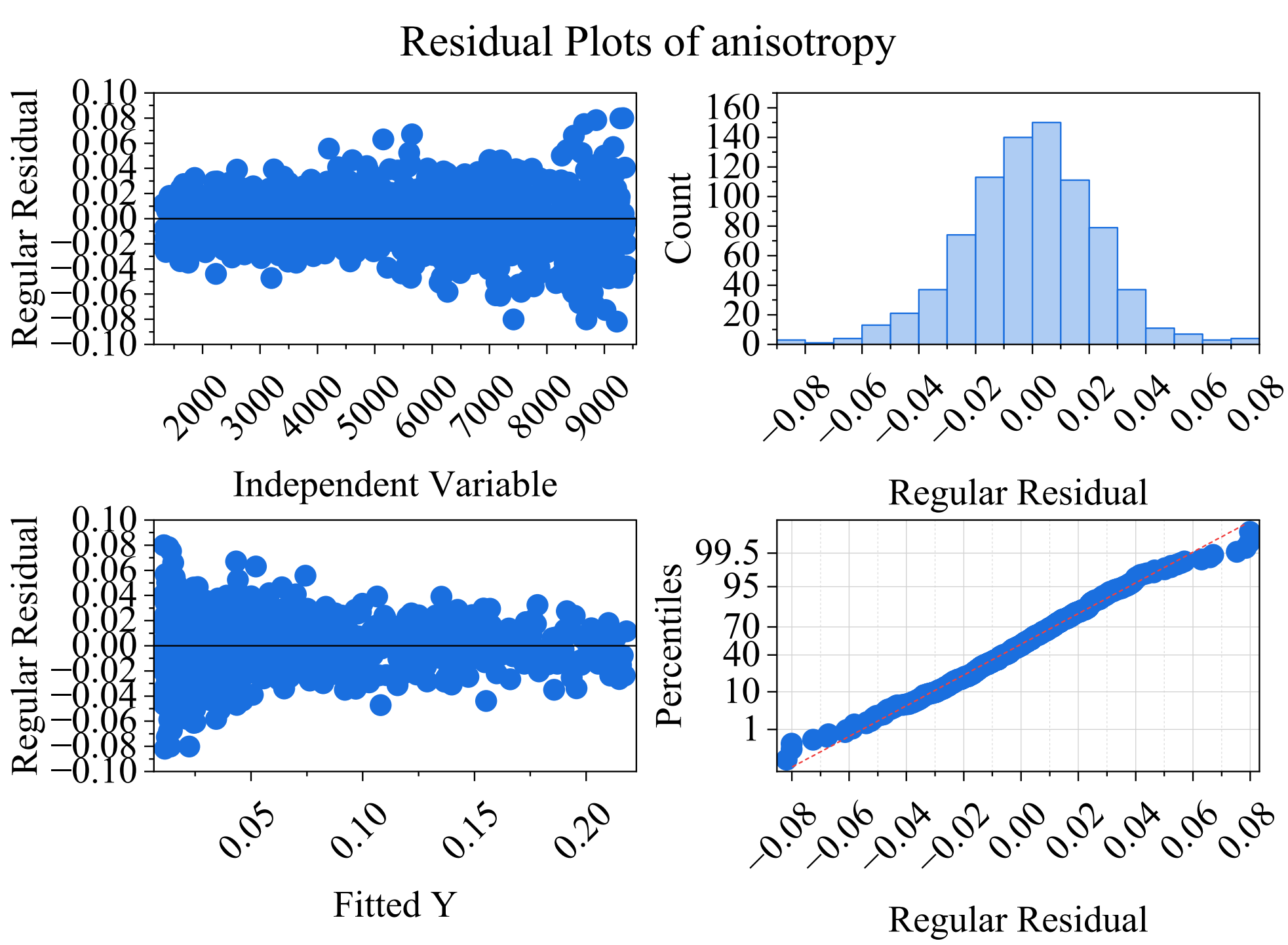

Figure S9. Nonlinear Curve Fit (ExpDecay1) for one data set of Perylene in

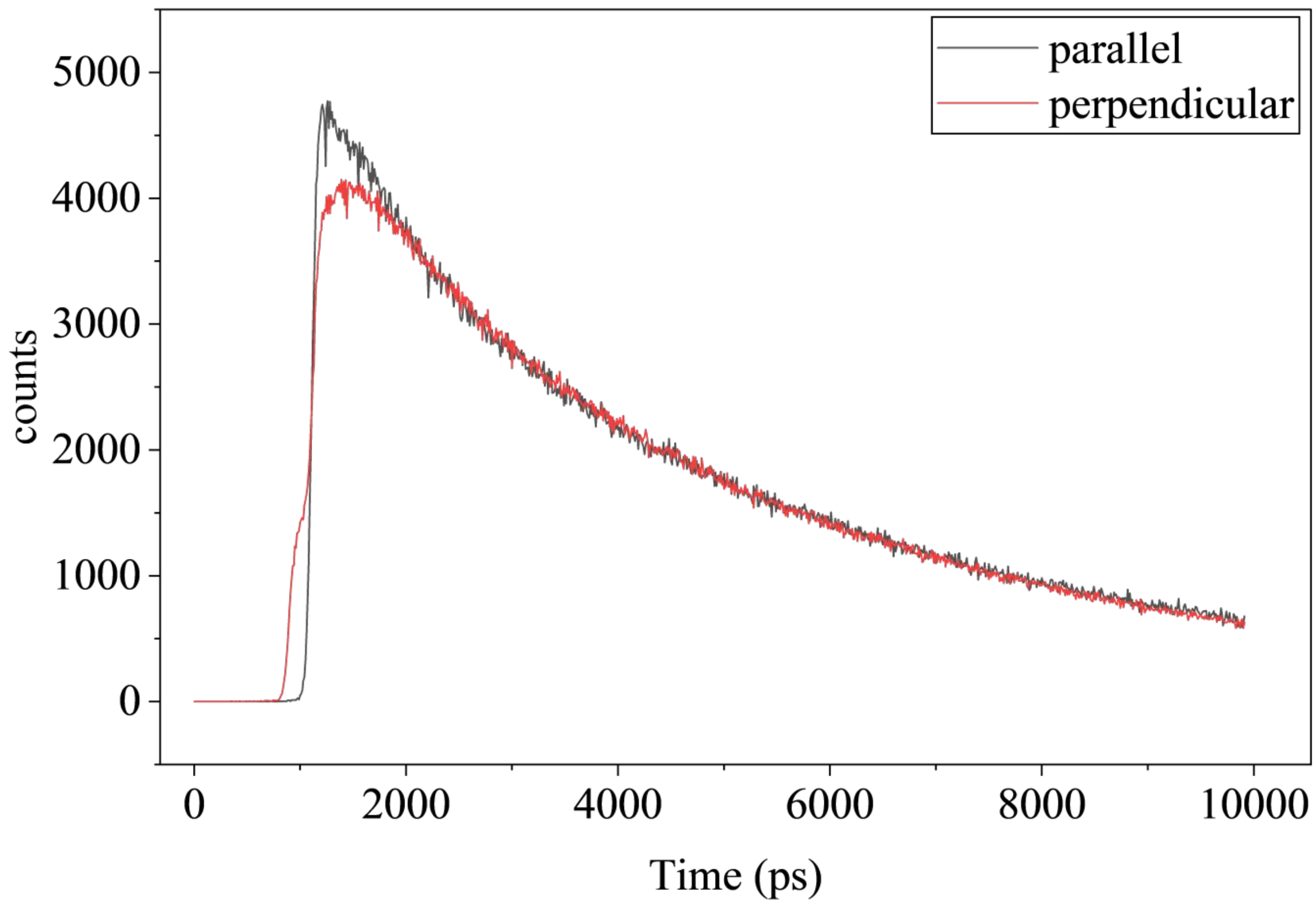

Nonlinear Curve Fit (ExpDecay1) (8/11/2026 13:30:33)
Parameters

		Value	Standard Error	t-Value	Prob>|t|	Dependency
M	y0	951.63989	20.90126	45.53027	9.89803E-230	0.96458
	x0	1210	0	--	--	0
	A1	12596.09006	17.55202	717.64332	0	0.72319
	t1	3510.07054	16.85695	208.22685	0	0.95307

Reduced Chi-sqr = 13135.7839888
COD(R^2) = 0.99852742706709
Iterations Performed = 2
Total Iterations in Session = 2
Fit converged. Chi-Sqr tolerance value of 1E-9 was reached.
Some parameter values were fixed.
Standard Error was scaled with square root of reduced Chi-Sqr.

Statistics

	M
Number of Points	849
Degrees of Freedom	846
Reduced Chi-Sqr	13135.78399
Residual Sum of Squares	1.11129E7
R-Square (COD)	0.99853
Adj. R-Square	0.99852
Fit Status	Succeeded(100)

Fit Status Code :
100 : Fit converged. Chi-Sqr tolerance value of 1E-9 was reached.

Summary

	y0		x0		A1		t1		Statistics	
	Value	Standard Error	Value	Standard Error	Value	Standard Error	Value	Standard Error	Reduced Chi-Sqr	Adj. R-Square
M	951.63989	20.90126	1210	0	12596.09006	17.55202	3510.07054	16.85695	13135.78399	0.99852

ANOVA

		DF	Sum of Squares	Mean Square	F Value	Prob>F
M	Regression	2	7.53546E9	3.76773E9	286829.32588	<0.0001
	Residual	846	1.11129E7	13135.78399		
	Uncorrected Total	849	3.24301E10			
	Corrected Total	848	7.54657E9			

M: At the 0.05 level, the fitting function is significantly better than the function y=constant.

Figure S10. Fitted Curves and Residuals Plots

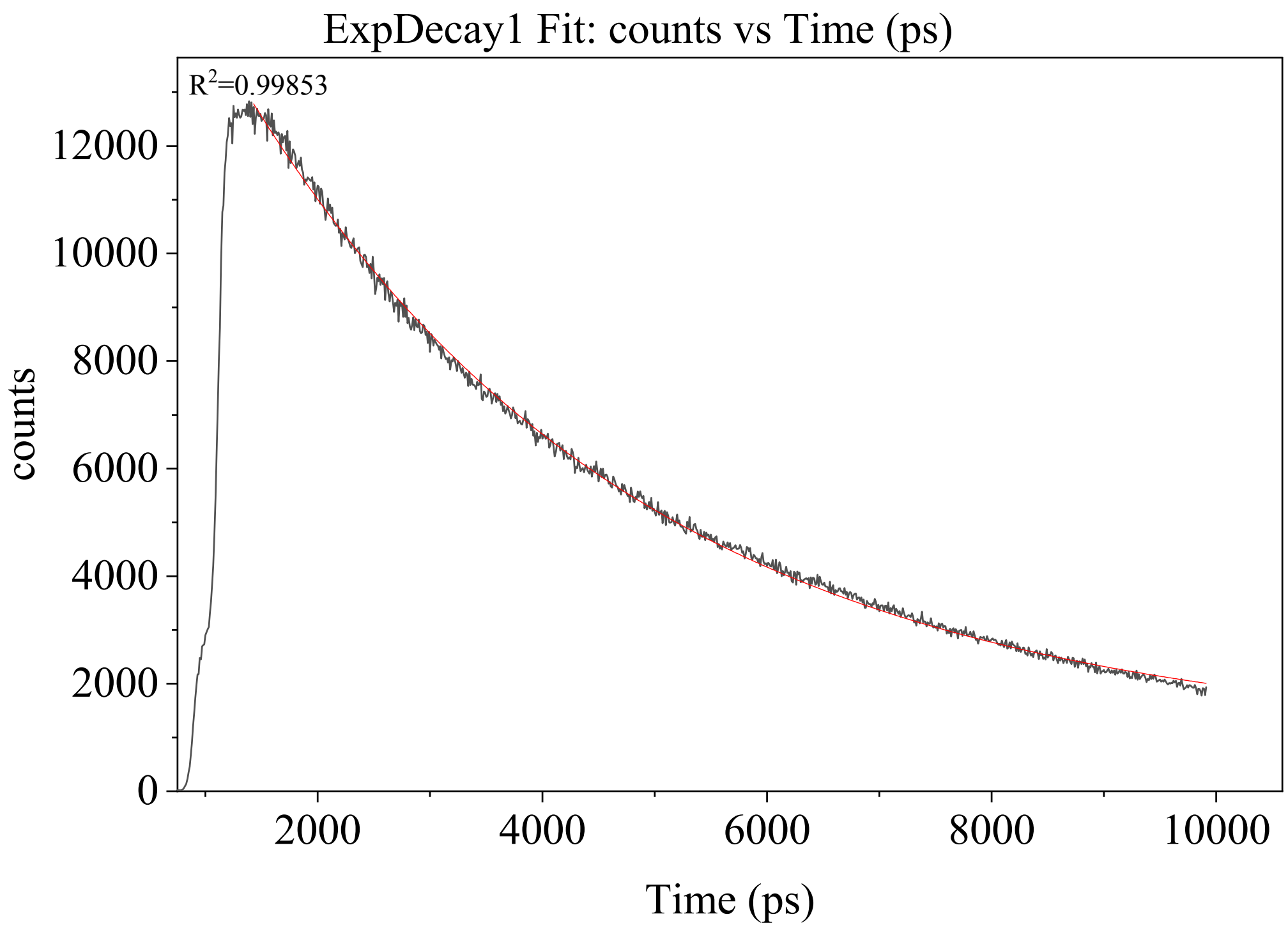

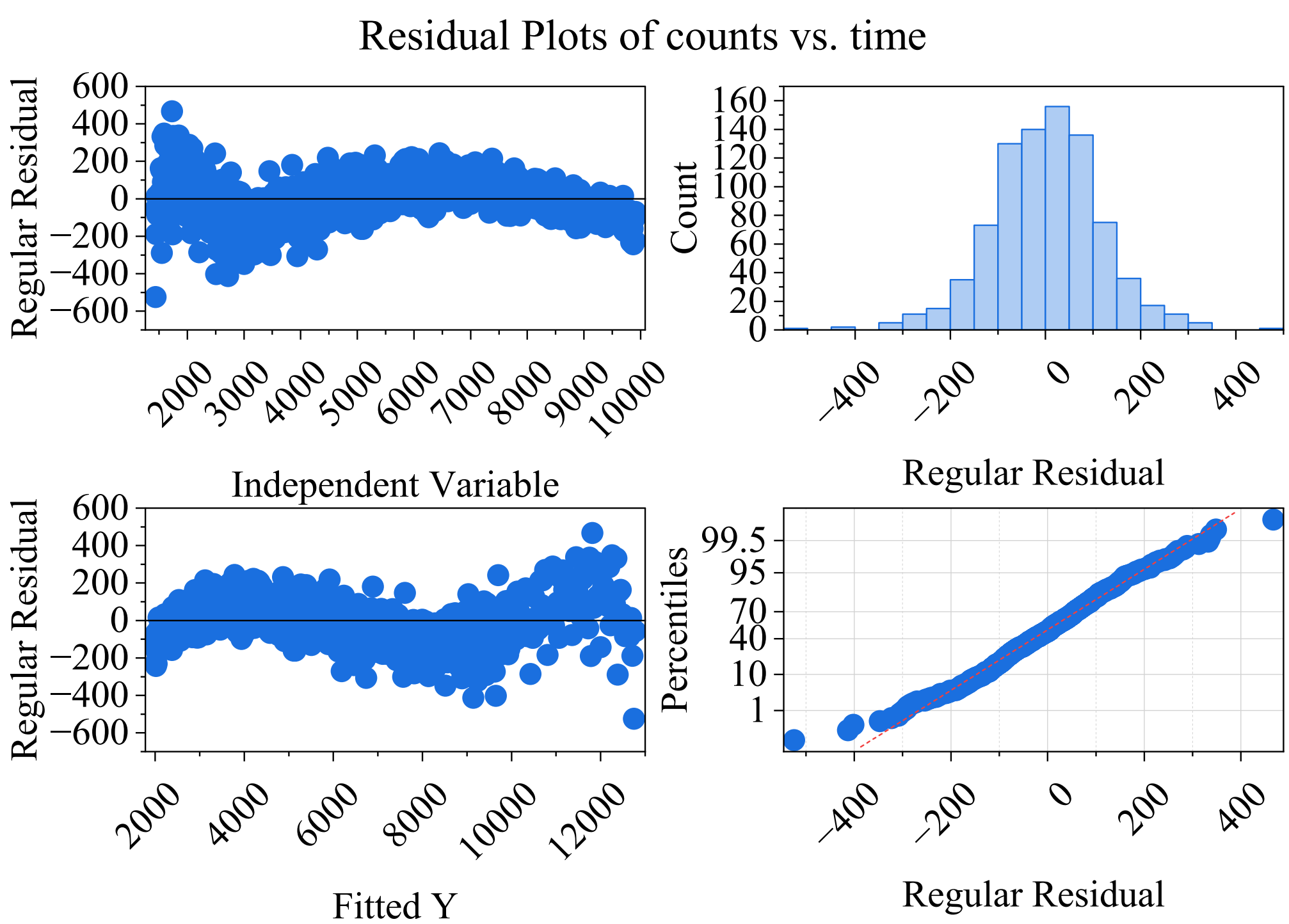

Table S10. Fit of perylene anisotropy decay data

Nonlinear Curve Fit (ExpDecay1)

Parameters

		Value	Standard Error	t-Value	Prob>|t|	Dependency
N	y0	0	0	--	--	0
	x0	1210	0	--	--	0
	A1	0.06802	0.00357	19.03037	1.77837E-66	0.36747
	t1	293.70432	24.55378	11.96168	2.29489E-30	0.36747

Reduced Chi-sqr = 1.50586304752E-4
COD(R^2) = 0.38221351234672
Iterations Performed = 5
Total Iterations in Session = 5
Fit converged. Chi-Sqr tolerance value of 1E-9 was reached.
Some parameter values were fixed.
Standard Error was scaled with square root of reduced Chi-Sqr.

Statistics

	N
Number of Points	772
Degrees of Freedom	770
Reduced Chi-Sqr	1.50586E-4
Residual Sum of Squares	0.11595
R-Square (COD)	0.38221
Adj. R-Square	0.38141
Fit Status	Succeeded(100)

Fit Status Code :
100 : Fit converged. Chi-Sqr tolerance value of 1E-9 was reached.

Summary

	y0		x0		A1		t1		Statistics	
	Value	Standard Error	Value	Standard Error	Value	Standard Error	Value	Standard Error	Reduced Chi-Sqr	Adj. R-Square
N	0	0	1210	0	0.06802	0.00357	293.70432	24.55378	1.50586E-4	0.38141

ANOVA

		DF	Sum of Squares	Mean Square	F Value	Prob>F
N	Regression	2	0.08622	0.04311	286.27396	<0.0001
	Residual	770	0.11595	1.50586E-4		
	Uncorrected Total	772	0.20217			
	Corrected Total	771	0.18769			

N: At the 0.05 level, the fitting function is significantly better than the function y=0.

Figure S11. Fitted Curves and Residuals Plots

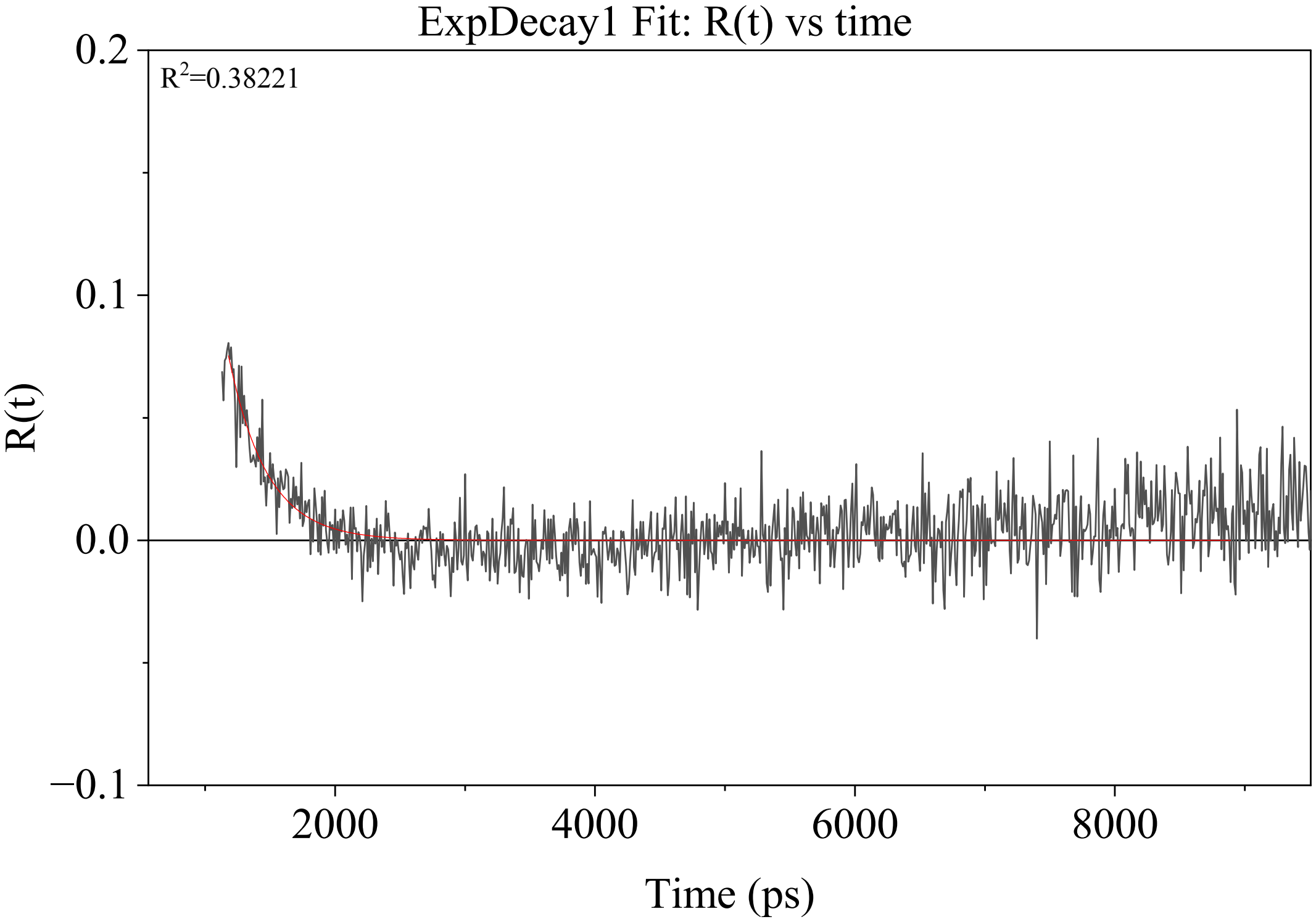

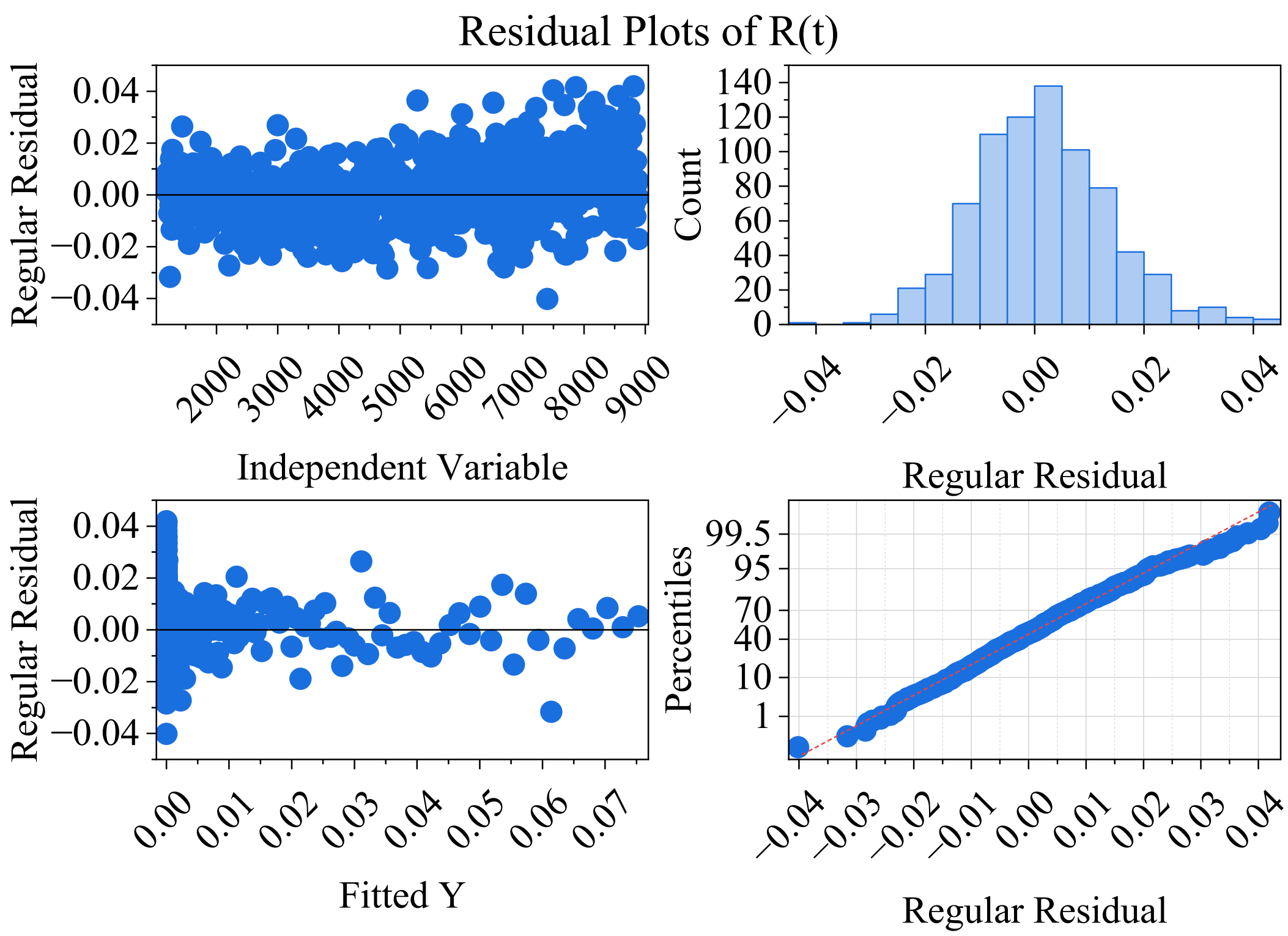

Supplement: Supplementary file 1 [file jp6c03764_si_001.pdf]
